# ambr™ Mini-bioreactor as a high-throughput tool for culture process development to accelerate transfer to stainless steel manufacturing scale: comparability study from process performance to product quality attributes

**DOI:** 10.1186/1753-6561-9-S9-P78

**Published:** 2015-12-14

**Authors:** Frédéric Delouvroy, Gaetan Siriez, An-Vy Tran, Larissa Mukankurayija, Nadine Kochanowski, Laetitia Malphettes

**Affiliations:** 1 Upstream Process Sciences, Biotech Sciences, UCB Pharma S.A., Chemin du Foriest, Braine l'Alleud, Belgium

## Background

Enhancing throughput of bioprocess development has become increasingly important to rapidly screen and optimize cell culture process parameters. With increasing timeline pressures to get therapeutic candidates into the clinic, resource intensive approaches such as the use of shake flasks and bench-top bioreactors may limit the design space for experimentation to yield highly productive processes. The need to conduct large numbers of experiments has resulted in the use of miniaturized high-throughput (HT) technology for bioprocess development. One such high-throughput system is the ambr™ platform, a robotically driven, mini-bioreactor system developed by TAP-Sartorius.

In this study we assessed and compared the performance parameters of ambr™ mini-bioreactor runs to 2L glasses , 80L and 400L stainless steel bioreactors using a CHO cell line producing a recombinant monoclonal antibody. The daily parameters monitored during the cultures were cell growth and cell viability, offline pH and dissolved oxygen, metabolite profiles (glucose, lactate and ammonia) and monoclonal antibody titer. In addition, we compared the product quality attributes (high and low molecular weight species, charge variants) of the clarified cell culture fluid post Protein-A elution generated in the mini-bioreactor run to the larger manufacturing scales.

## Materials and methods

A genetically engineered Dihydrofolate Reductase (DHFR)-/ - DG44 Chinese Hamster Ovary (CHO) cell line with Methotrexate (MTX) as a selective agent, expressing a recombinant monoclonal antibody was used. Cells were cultivated for 14 days in a fed-batch mode in a chemically defined medium and fed according to process description.

Culture systems: Different bioreactor scales were used in this study : ambr™48 (TAP -Sartorius Biosystems), an automated system with 48 disposable microbioreactor vessels, 2L stirred tank glasses bioreactors with Biostat B-DCUII control systems (Sartorius Stedim), 80L and 400L stainless-steel bioreactors (Zeta).

Data was analysed using JMP statistical (SAS) program.

All the experiments were conducted using the same cell bank at the same cell age at bioreactor inoculation.

pH (7.0 +/- 0.2) was controlled using CO2 and base addition.

The scale independent factors (pH, DO set point, seeding density, temperature, culture duration, media and feed composition), were the same for all the scales.

The scale dependent factors (culture start volume, feed volumes) were linearly adapted. Agitation speed and aeration that were determined theoretically or though experiment.

Sampling plans and sample volumes were especially adapted to the ambr™ system to take into account the low bioreactor volume.

A miniaturised metabolite assay was developed to allow daily measurements glucose, ammonium, lactate and osmolality using low analyte volumes.

Viable Cell Density (VCD) and cell viability were measured using a ViCell® XR cell counter (Beckman Coulter) directly connected to the ambr™ system. Metabolites (glucose, lactate, ammonium) concentrations were measured by an enzymatic assay using a UV-method (R-Biopharm) for the ambr™ vessels and by a BioProfile Analyser 400 (Nova Biomedical) for larger scale bioreactors. For all the systems, pH measurement was obtained with a BioProfile pHOx pH/Gas Analyser (Nova Biomedical). And osmolality was obtained using a Osmometer (Advanced Instruments). Production titers were measured throughout the culture using an Octet QK (ForteBio) and after 14 days with protein A HPLC (Waters).

Product quality attributes (PQA) of the produced monoclonal antibodies were analysed as follows: Cell culture fluid samples were centrifuged and filtered to remove cell debris. The monoclonal antibodies were purified by ÄKTA-express (GE Healthcare) Protein-A purification. The neutralized eluate was used for product quality analysis. Charge variant analysis determined the relative percentage of acidic, basic and main isoform species of the mAb by means of an imaged capillary isoelectric focusing system (Protein Simple, iCE3) after a Carboxypeptidase B digestion. Size exclusion analysis method determined the percentage of aggregates, monomers and fragment levels by size exclusion chromatography (SE-UPLC).

## Results and discussion

To assess the capabilities of the ambr™ system to mimic traditional larger scale stirred tank bioreactors in terms of process performance, the 19 mini-bioreactors have been compared to 2L (n = 6), 80L (n = 3) and 400L (n = 3) bioreactors (Table 1).

**Table 1:** Number of bioreactors and batches performed at different scales

Scales	Number of bioreactors	Number of batches
ambr™	19	4
2L	6	3
80L	3	3
400L	3	3

Good reproducibility was obtained amongst the replicates intra-runs. Inter-runs, the different ambr™ bioreactor batches showed similar trends for cell viability. Nevertheless, a higher variability was observed for the VCD and the mAb titer. This might be due to the biological variability of the process. Although variability was observed, cells showed comparable mAb specific productivity between the different ambr™ batches. In terms of pH, and metabolite comparable profiles were obtained within the different mini-bioreactors ambr™ runs.

**Figure 1:**
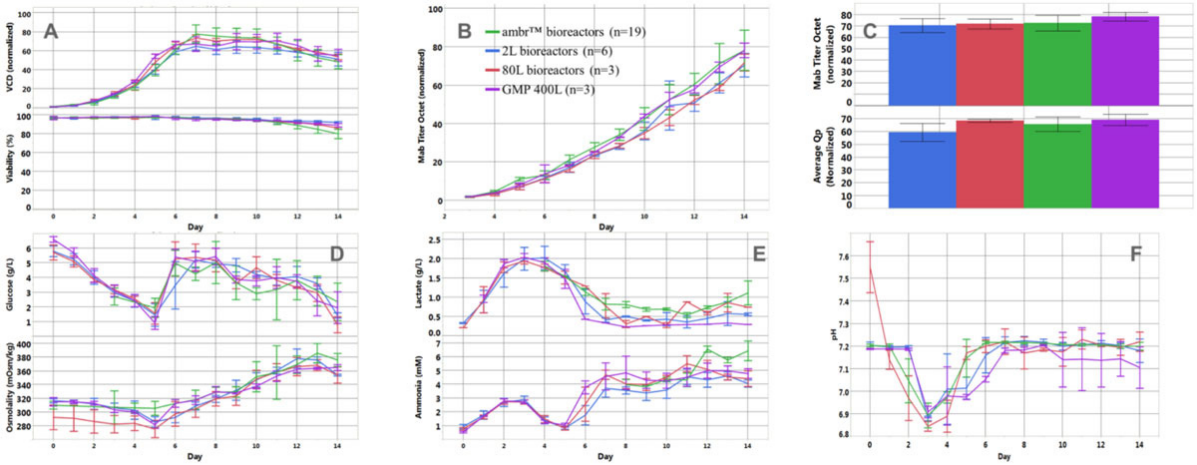
**Comparison between the different scales**. A: Viable cell density and viability profiles. B: mAb titer profile. C: mAb titer and average Qp value at day14. D: Osmolality and glucose profiles. E: Lactate and ammonium profiles. F: Offline pH profiles

Compare to larger scales (2L glasses, 80L and 400L stainless steel stirred tank bioreactors), cell growth, product titer, pH, and metabolites in ambr ™ mini-bioreactors displayed similar profiles to larger scales (Figure 1).

The product quality analysis showed comparable results between ambr™ and larger scale bioreactors (Figure not showed).

This study showed that for this antibody, ambr™ could successfully mimic larger scale bioreactors and give good predictive results. The equipment can be used as a high throughput platform for cell culture development. However, the scale down model developed in the mini-bioreactors might require fine tuning for each new biologic producing cell line.

The comparability in terms of process performance and product quality attribute between the 15mL ambr™ mini-bioreactors and the 400L stainless steel production bioreactors enables lean, fast and efficient process development minimising time to clinic.
